# Supplementary material for: The T-Cell Response to Type 2 Porcine Reproductive and Respiratory Syndrome Virus (PRRSV)
Source: Viruses. 2019 Aug 29;11(9):796. doi: 10.3390/v11090796 (PMC6784018; doi:10.3390/v11090796)
Supplement: Supplementary file 1 [file viruses-11-00796-s001.pdf]

Antigen	Clone	Isotype	Fluorochrome	Labeling strategy	Primary Ab source	2nd Ab source
CD3	PPT3	IgG1	FITC	Directly conjugated	Southern Biotech	-
CD4	74-12-4	IgG2b	PerCP-Cy5.5	Directly conjugated	BD Biosciences	-
CD8α	76-2-11	IgG2a	Brilliant Violet 480	Secondary antibody	BD Biosciences	Jackson Immunoresearch
TCR-$\gamma\delta$	PGBL22A	IgG1	Alexa Fluor 647	Directly conjugated	Kingfisher	Invitrogen
CD172a	74-22-15	IgG1	Alexa Fluor 680	Directly conjugated	BEI Resources	Invitrogen
CD21a	BB6-11C9.6	IgG1	Brilliant Violet 605	Biotin-streptavidin	Novusbio	Biolegend
CD21b	B-ly4	IgG1	Brilliant Violet 421	Directly conjugated	BD Biosciences	-
CD27	B30C7	IgG1	PE	Directly conjugated	BIO-RAD	
Live / Dead	-	-	Near Infra-red	-	Invitrogen	-

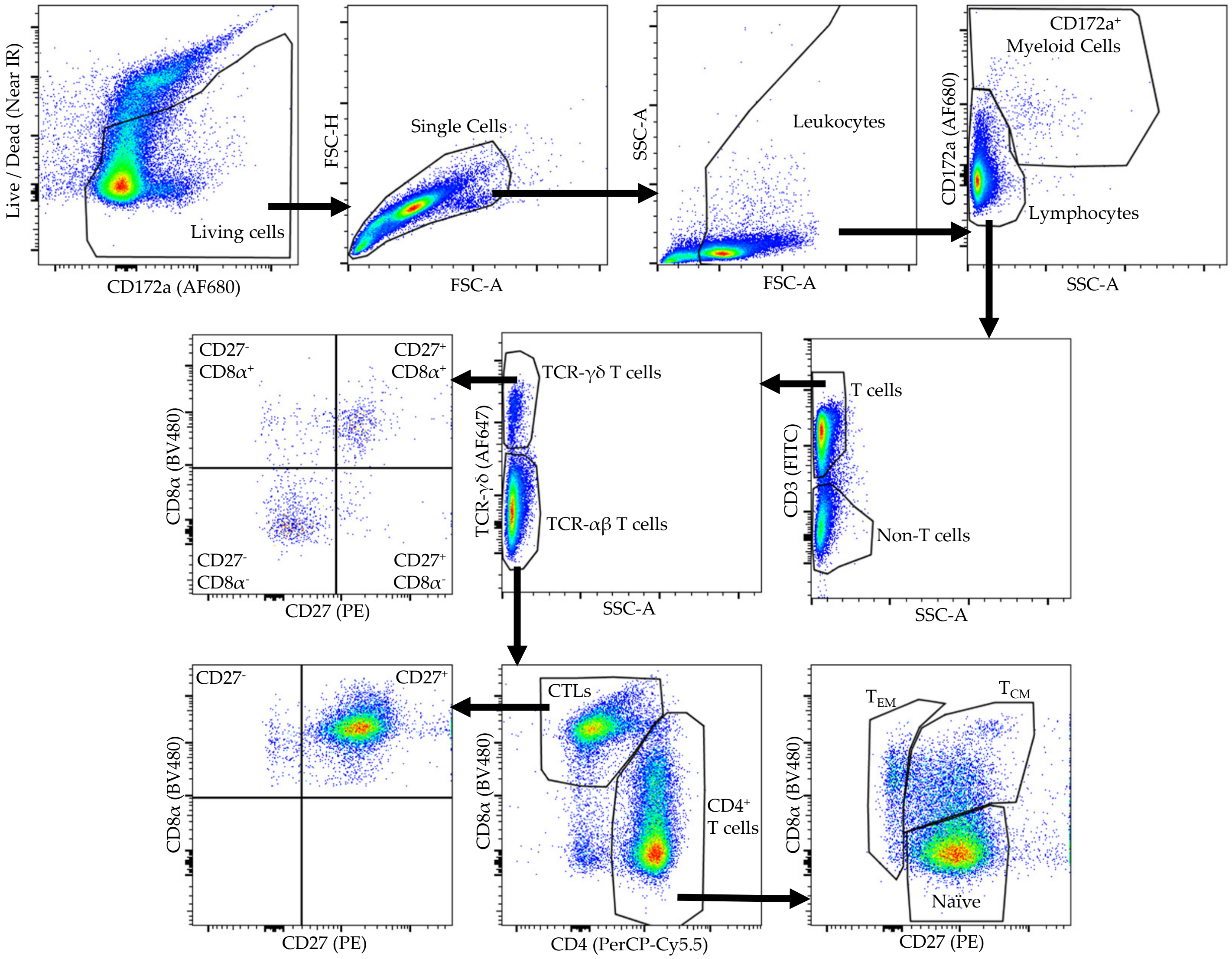

* Gating hierarchy from MOCK animal, tracheobronchial lymph node

Supplemental Figure 1. Necropsy tissue 9-color staining panel and gating hierarchy

Antigen	Clone	Isotype	Fluorochrome	Labeling strategy	Primary Ab source	2nd Ab source
CD3	PPT3	IgG1	FITC	Directly conjugated	Southern Biotech	-
CD4	74-12-4	IgG2b	PerCP-Cy5.5	Directly conjugated	BD Biosciences	-
CD8α	76-2-11	IgG2a	Brilliant Violet 421	Secondary antibody	BD Biosciences	Jackson Immunoresearch
TCR-γδ	PGBL22A	IgG1	Alexa Fluor 647	Directly conjugated	Kingfisher	Invitrogen
Foxp3	FJK-16s	IgG2a	PE	Directly conjugated	Invitrogen	-
Live / Dead	-	-	Near Infra-red	-	Invitrogen	-

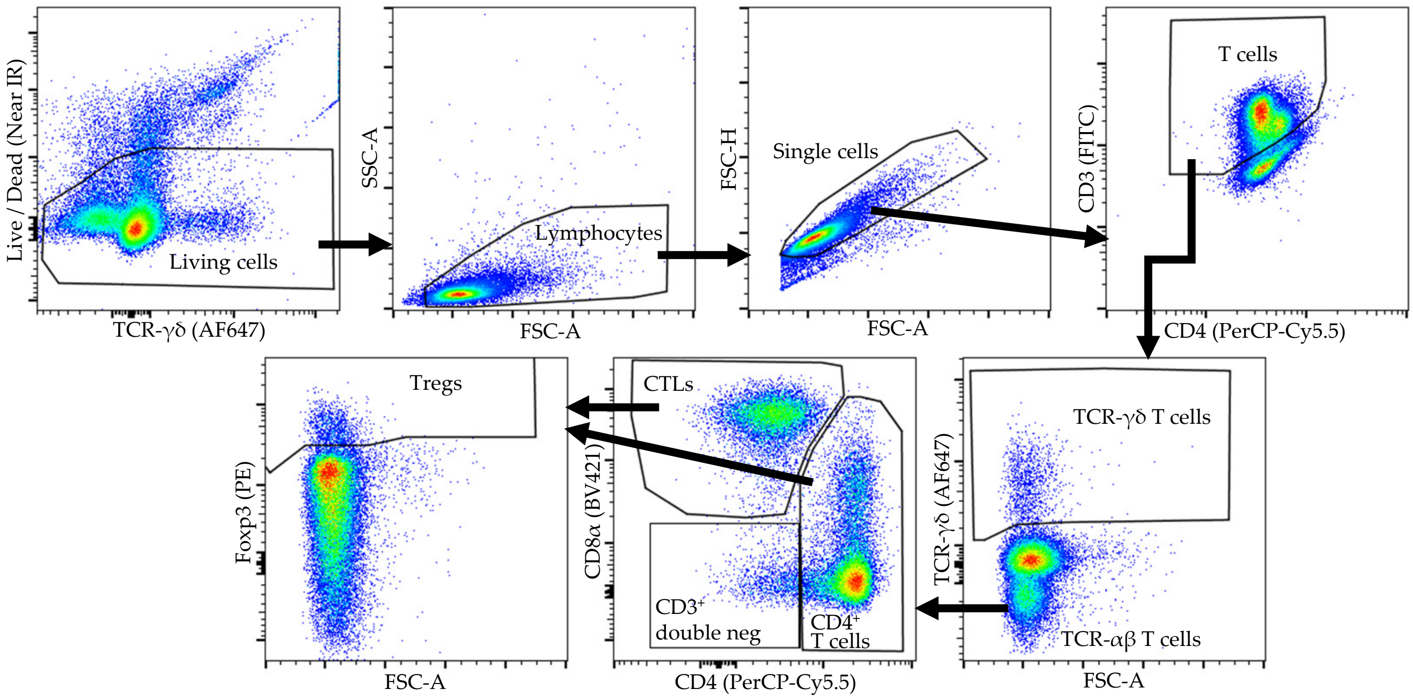

* Gating hierarchy from MOCK animal, tracheobronchial lymph node

Supplemental Figure 2. Necropsy tissue Treg staining panel and gating hierarchy

Antigen	Clone	Isotype	Fluorochrome	Labeling strategy	Primary Ab source	2nd Ab source
CD14	Tuek4	IgG2a	FITC	Directly conjugated	ThermoFisher	-
CD163	2a10-11	IgG1	PE	Directly conjugated	ThermoFisher	-
CD172a	74-22-15A	IgG2b	Brilliant Violet 421	Secondary antibody	Kingfisher	Jackson Immunoresearch
PRRSV	SR30-A	IgG1	Alexa Fluor 647	Directly conjugated	RTI	Invitrogen
Live / Dead	-	-	Near Infra-red	-	Invitrogen	-

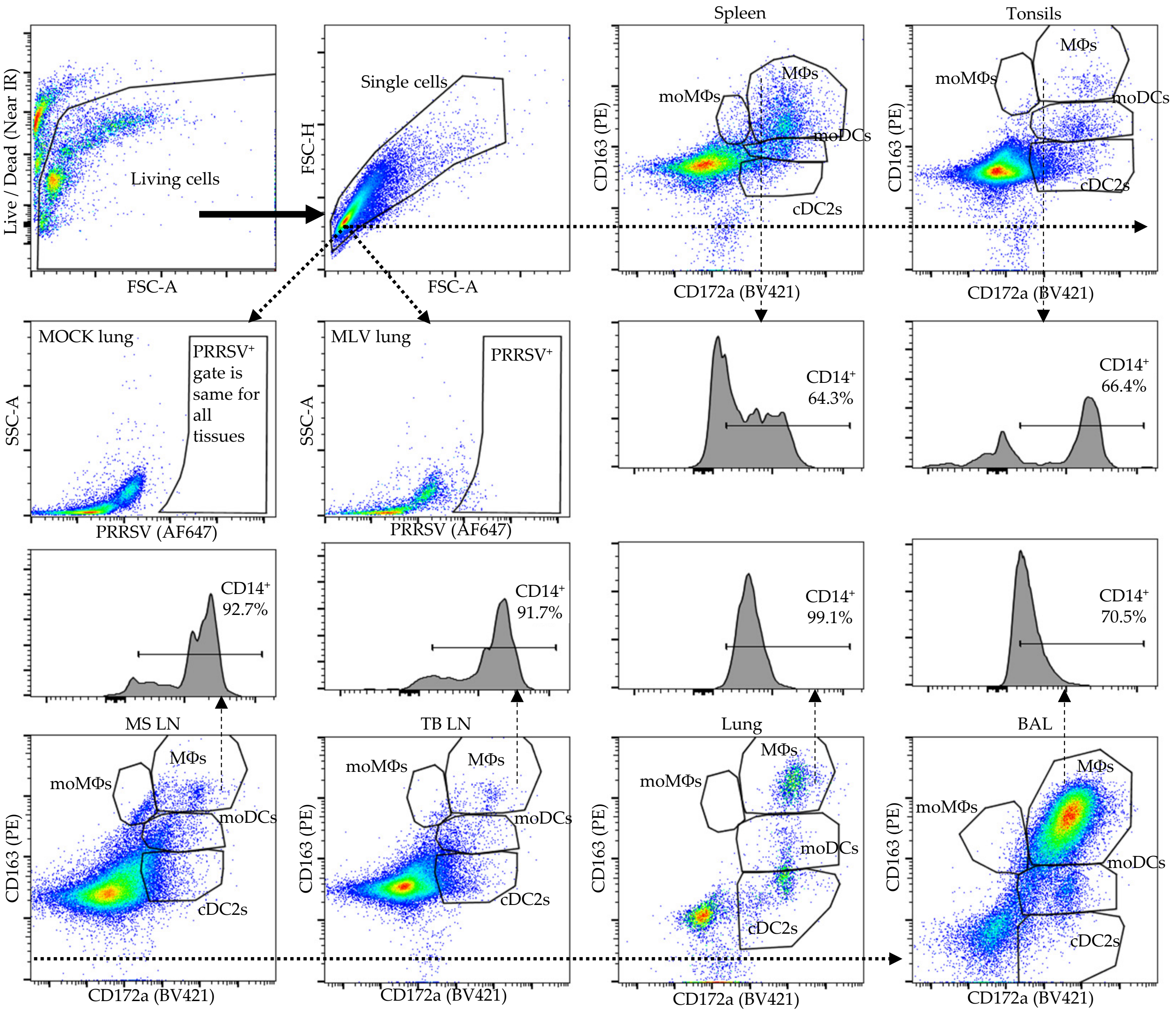

MΦ = Macrophage   DC = Dendritic cell   mo = monocytes differentiating into MΦ or DCs

* CD163 and CD172a gating hierarchy from MLV pig; PRRSV+ gating hierarchy from MOCK and MLV pig

Supplemental Figure 3. Necropsy tissue PRRSV staining panel and gating hierarchy

Antigen	Clone	Isotype	Fluorochrome	Labeling strategy	Primary Ab source	2nd Ab source
CD3	PPT3	IgG1	FITC	Directly conjugated	Southern Biotech	-
CD4	74-12-4	IgG2b	Brilliant Violet 421	Secondary antibody	BEI Resources	Jackson Immunoresearch
CD8α	76-2-11	IgG2a	PE-Cy5.5	Biotin-streptavidin	Southern Biotech	Southern Biotech
CCR7	3D12	rIgG2a	Brilliant Violet 480	Directly conjugated	BD Biosciences	-
IFN-γ	P2G10	IgG1	PE	Directly conjugated	BD Biosciences	-
TNF-α	Mab11	IgG1	Brilliant Violet 605	Directly conjugated	Biolegend	-
IL-2	PG164A	IgG2a	Alexa Fluor 647	Secondary antibody	Invitrogen	Invitrogen
Live / Dead	-	-	Near Infra-red	-	Invitrogen	-

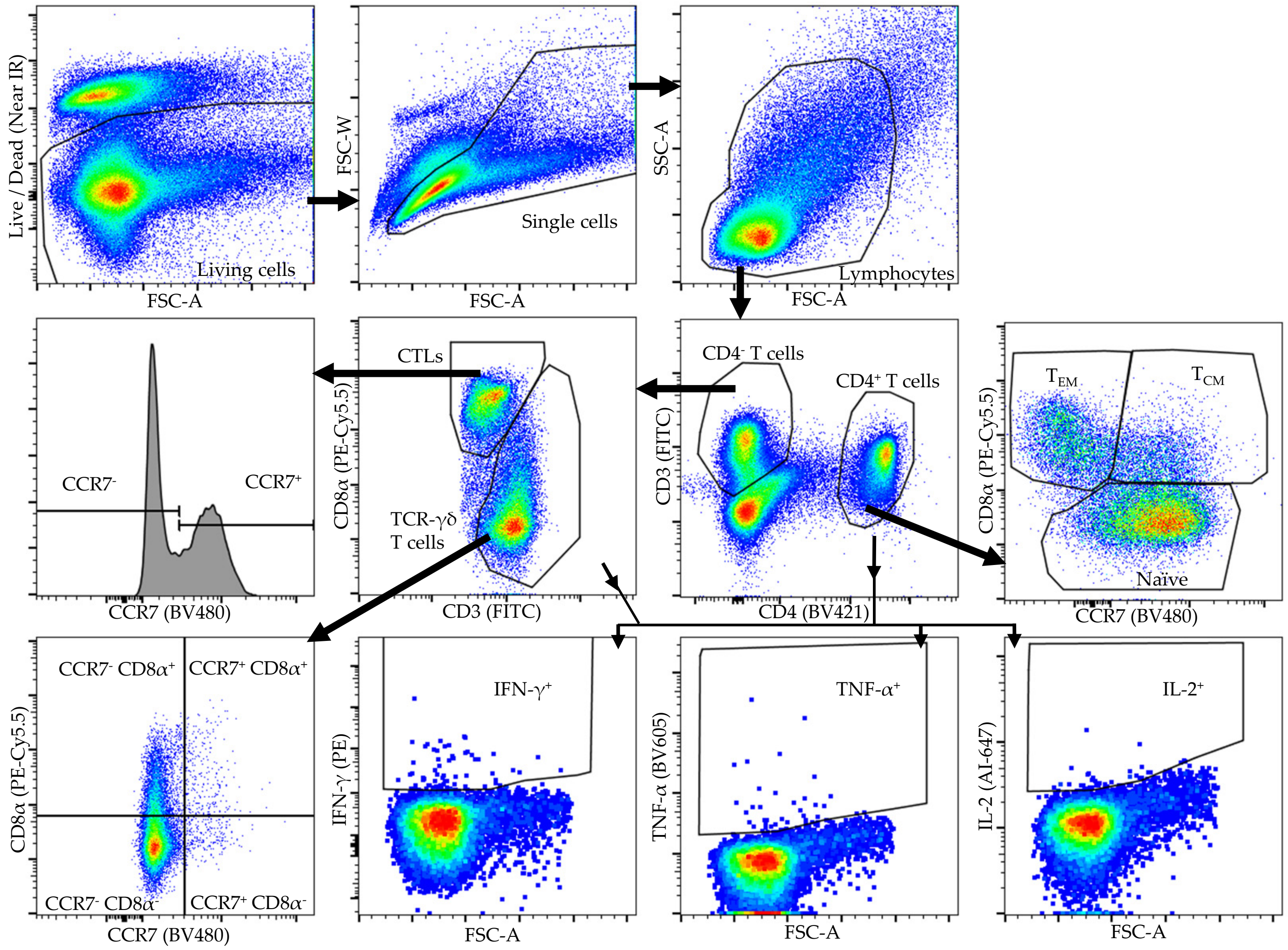

* Gating hierarchy from LP animal stimulated with strain 1-3-4

Supplemental Figure 4. PBMC Intracellular cytokine staining panel and gating hierarchy

Antigen	Clone	Isotype	Fluorochrome	Labeling strategy	Primary Ab source	2nd Ab source
CD3	PPT3	IgG1	FITC	Directly conjugated	Southern Biotech	-
CD4	74-12-4	IgG2b	Brilliant Violet 480	Secondary antibody	BEI Resources	Jackson Immunoresearch
CD8α	76-2-11	IgG2a	Brilliant Violet 605	Biotin-streptavidin	Southern Biotech	Biolegend
TCR-γδ	PGBL22A	IgG1	Alexa Fluor 647	Directly conjugated	Kingfisher	Invitrogen
CCR7	3D12	rIgG2a	Brilliant Blue 700	Directly conjugated	BD Biosciences	-
Foxp3	FJK-16s	IgG2a	PE	Directly conjugated	Invitrogen	-
Prolif-eration	-	-	CellTrace™ Violet	-	Invitrogen	-
Live / Dead	-	-	Near Infra-red	-	Invitrogen	-

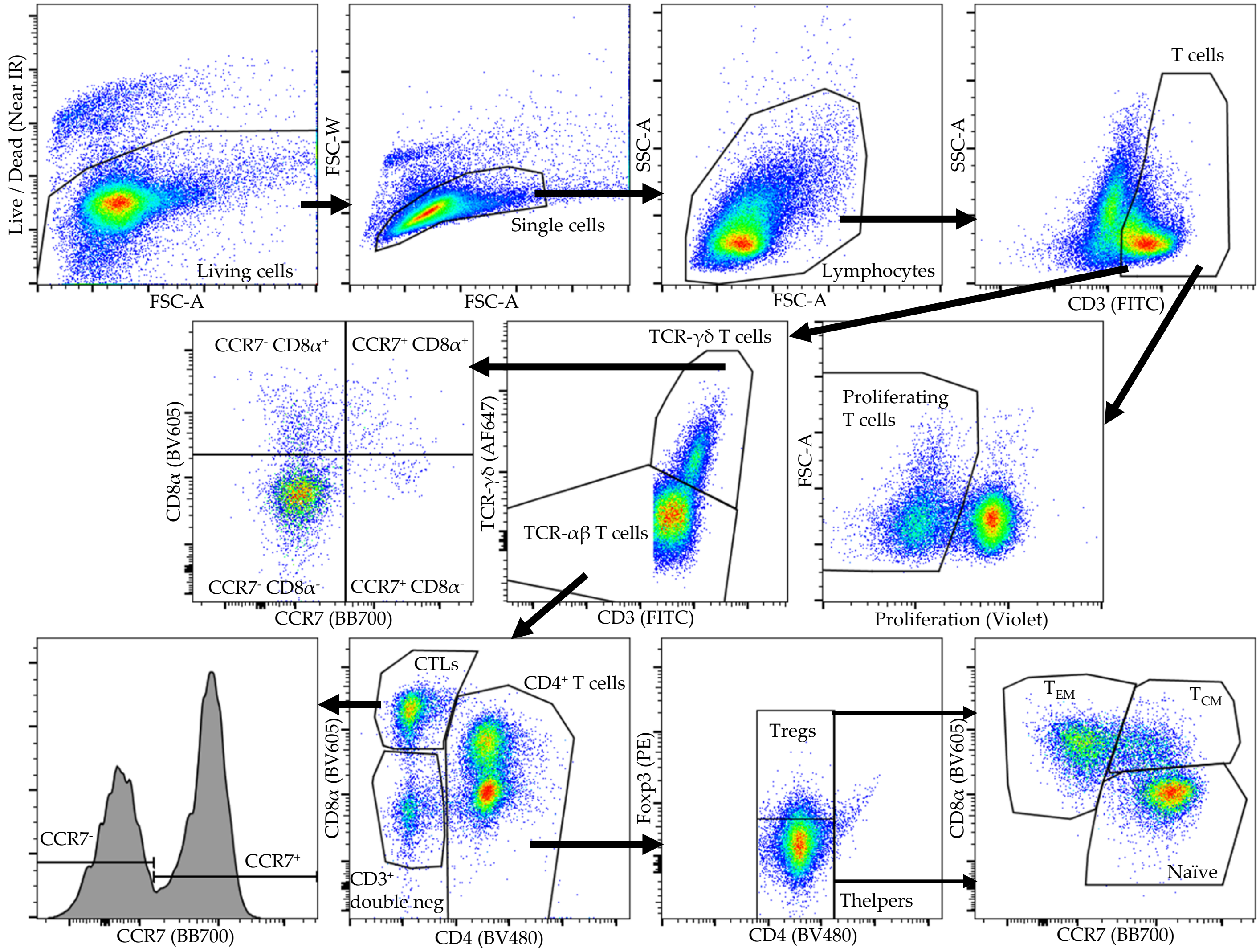

* Gating hierarchy from HP animal stimulated with strain 1-7-4

Supplemental Figure 5. PBMC proliferation staining panel and gating hierarchy

Antigen	Clone	Isotype	Fluorochrome	Labeling strategy	Primary Ab source	2nd Ab source
CD3	PPT3	IgG1	FITC	Directly conjugated	Southern Biotech	-
CD4	74-12-4	IgG2b	PerCP-Cy5.5	Directly conjugated	BD Biosciences	-
CD8α	76-2-11	IgG2a	PE	Directly conjugated	BD Biosciences	-
TCR-γδ	PGBL22A	IgG1	Alexa Fluor 647	Directly conjugated	Kingfisher	Invitrogen
Foxp3	FJK-16s	IgG2a	PE-Cy7	Directly conjugated	Invitrogen	-
Prolif-eration	-	-	CellTrace™ Violet	-	Invitrogen	-
Live / Dead	-	-	Near Infra-red	-	Invitrogen	-

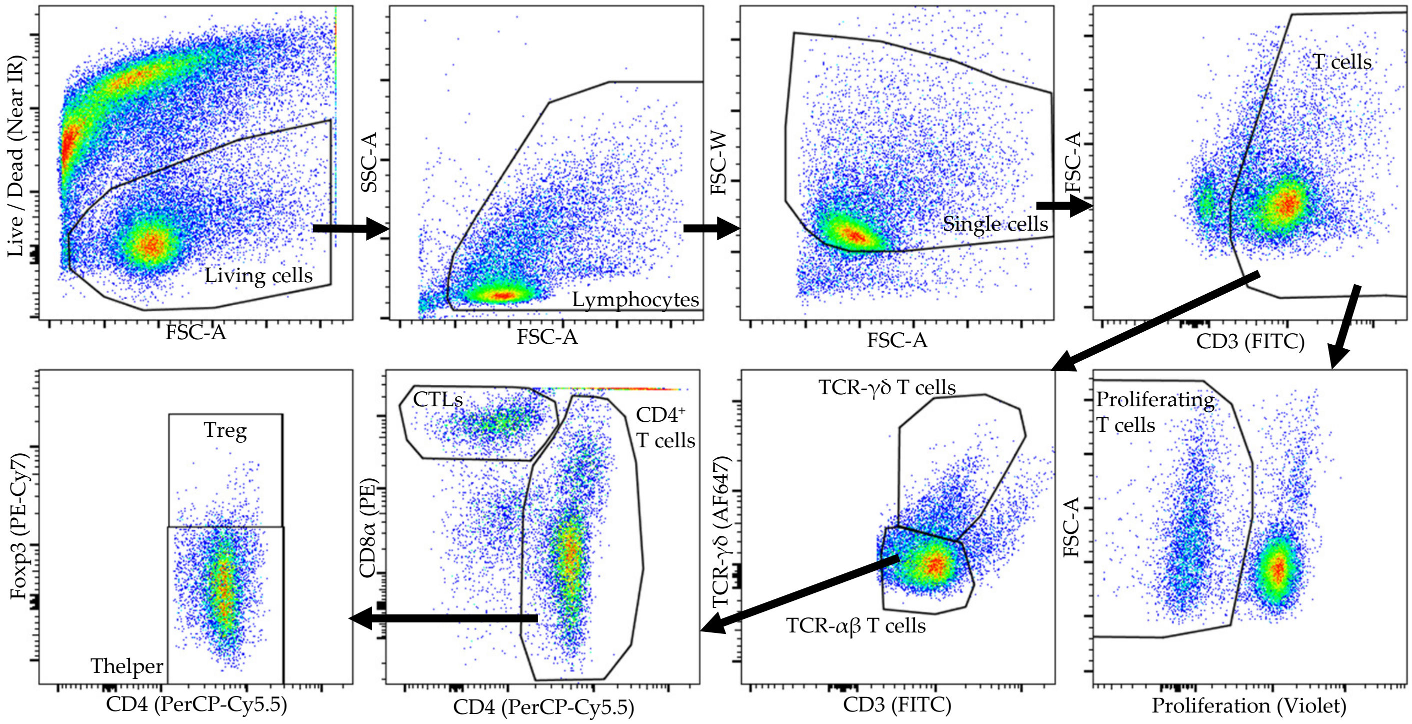

* Gating hierarchy from LP animal stimulated with strain 1-3-4

Supplemental Figure 6. Necropsy lymph node proliferation panel and gating hierarchy
